# A rare co-existence of histiocytic necrotizing lymphadenitis with metastatic papillary thyroid carcinoma and review of the literature

**DOI:** 10.1186/s13000-024-01441-0

**Published:** 2024-01-13

**Authors:** Jing Liu, Lei Jiang, Guohua Yu, Guimei Qu, Li Cai

**Affiliations:** 1 Weifang Medical University, Weifang, China; 2 Department of Pathology, The Affiliated Yantai Yuhuangding Hospital of Qingdao University, Yantai, China

**Keywords:** Histiocytic necrotizing lymphadenitis, Metastatic papillary thyroid carcinoma, Coexistent disease

## Abstract

Histiocytic necrotizing lymphadenitis (HNL) is a benign, self-limiting disease that is rare clinically. The coexistence of HNL and tumor is rarer. We report a male patient who was preoperatively diagnosed with papillary thyroid carcinoma with cervical lymph nodes metastasis, and the postoperative pathological examination showed histiocytic necrotizing lymphadenitis combined with metastatic papillary thyroid carcinoma in the same single lymph node. More interestingly, Epstein‒Barr virus was positive in these lymph nodes by in situ hybridization. This may suggest a trigger for the coexistence of the two diseases.

## Introduction

Histiocytic necrotizing lymphadenitis (HNL) is a self-limiting disease of unknown cause, also known as Kikuchi-Fujimoto disease (KFD). It was first reported in 1972 by KIKUCHI and FUJIMOTO et al. [1]. HNL is a relatively uncommon benign lymph node enlargement, a self-limiting disease that usually occurs in Asian women in their 20 and 30 s. Some studies have shown a prevalence of nearly 1:2 in men and women. The most common symptoms are enlarged lymph nodes in the neck with tenderness and fever. The etiology of HNL is unclear. The association of HNL and malignancy is also seldom discussed.

The coexistence of HNL and tumor is extremely rare. Herein, we report a case of metastatic papillary thyroid carcinoma coexistent with histiocytic necrotizing lymphadenitis in the same lymph node.

## Case report

**Fig. 1:**
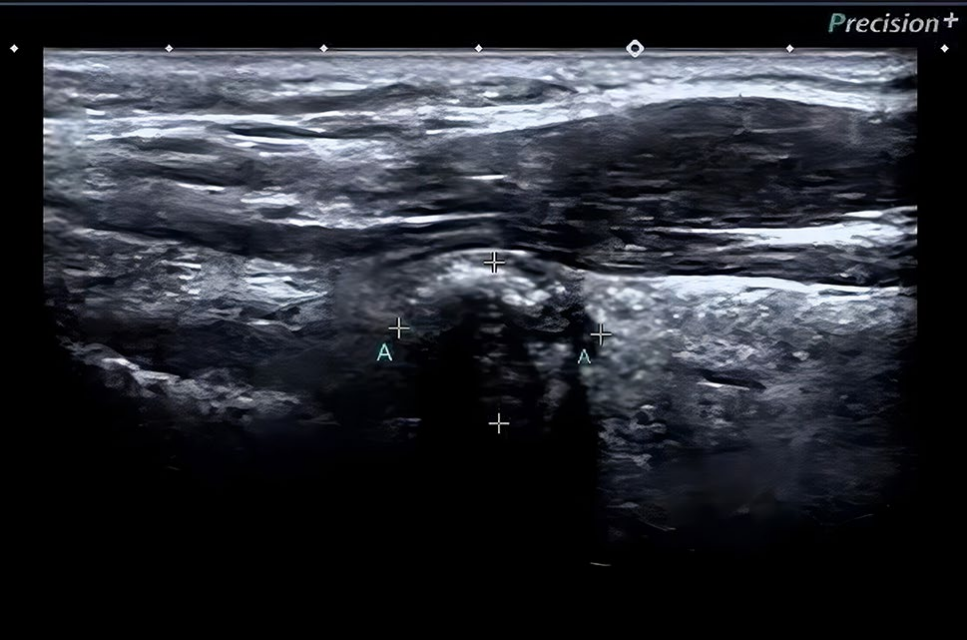
Ultrasound showed a 1.3 × 1.0 cm hypoechoic nodule with a faint border in the upper pole of the right lobe of the thyroid gland. (A: Thyroid mass)

**Fig. 2:**
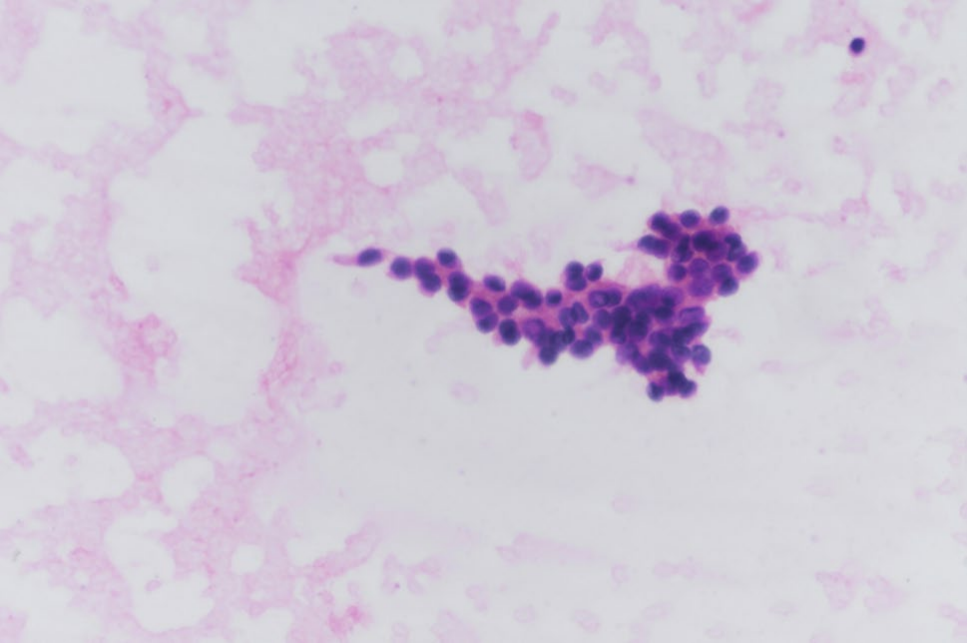
FNA smear for the right lobe of the thyroid gland: Microscopy showed that the follicular epithelium was arranged in a papillary structure, with enlarged nuclei, irregular thickening of nuclear membranes, and nuclear grooves and pseudoinclusion bodies

**Fig. 3:**
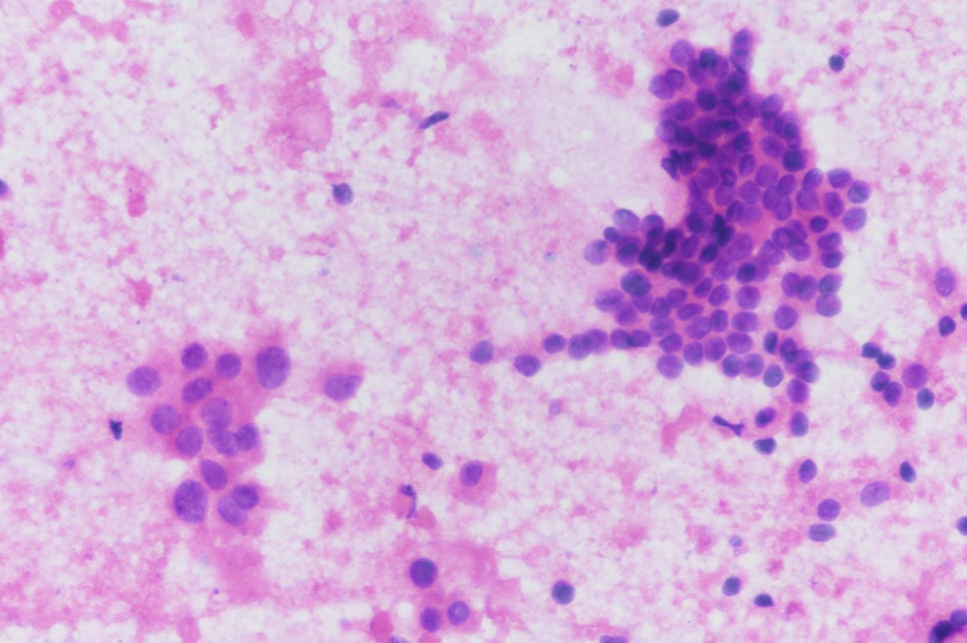
FNA smear of the right cervical lymph node shows that follicular epithelioid cells are arranged in lamellar nests, and nuclei are enlarged and crowded. Nuclear sulci and internal inclusion bodies can be seen. (hematoxylin and eosin, original magnification, x 400)

A 48-year-old man was admitted to the hospital with a diagnosis of papillary thyroid carcinoma confirmed by fine needle aspiration of the thyroid after 20 days of physical examination. Ultrasound examination of the thyroid showed that a hypoechoic nodule was detected in the upper pole of the right lobe of the thyroid gland, approximately 1.3 × 1.0 cm, with regular morphology, aspect ratio < 1, fuzzy border, uneven internal echogenicity, and multiple dotted strong echogenicity with rear echo attenuation. Color Doppler flow imaging (CDFI): no significant signal was observed. Enlarged lymph nodes were seen in the II-IV region of the right neck. The largest lymph node was about 2.1 × 1.4 cm, with full morphology, clear borders, thickened cortex, and disappearance of lymphatic portal structures, and scattered strong echogenicity was detected in some of the nodes. CDFI: Blood flow signal was visible in the lymph nodes. A slightly larger lymph node was detected in the II-IV area of the left neck, about 1.0 × 0.5 cm. The findings were “nodule in the right lobe of the thyroid: Thyroid Imaging Reporting and Data System (TI-RADS) category 4a nodule in the middle of the right lobe, multiple enlarged lymph nodes in the right side of the neck; slightly enlarged lymph nodes in the left side of the neck” (Fig. 1). The diagnosis of fine needle aspiration of the thyroid and lymph node were shown: (right thyroid) Bethesda grade VI, papillary thyroid carcinoma; (right cervical lymph node) metastatic carcinoma, consistent with metastatic papillary thyroid carcinoma. (Figs. 2 and 3). For further diagnosis and treatment, he was admitted on August 20, 2022. After completing the laboratory and other related examinations, thyroid surgery was performed.

**Fig. 4:**
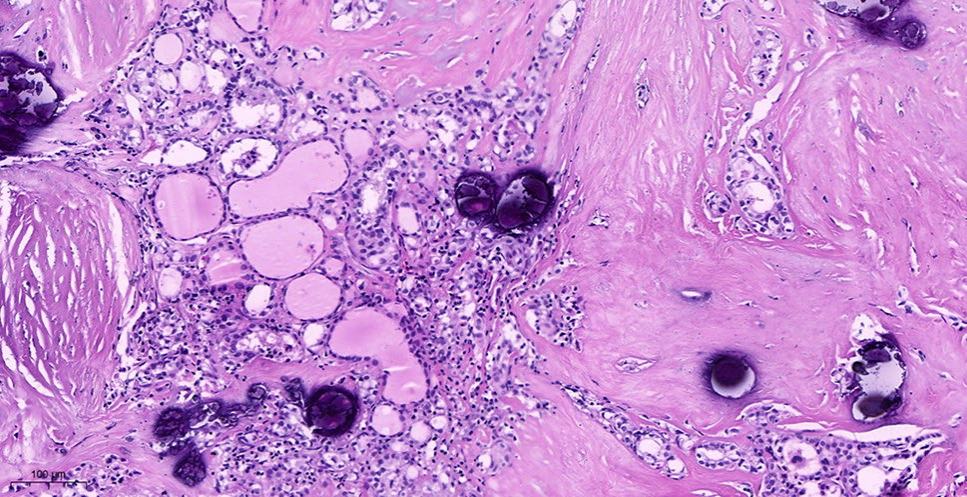
Postoperative paraffin of right thyroid section: Papillary carcinoma of the right thyroid with extensive psammoma body (hematoxylin and eosin, original magnification, x 100)

**Fig. 5:**
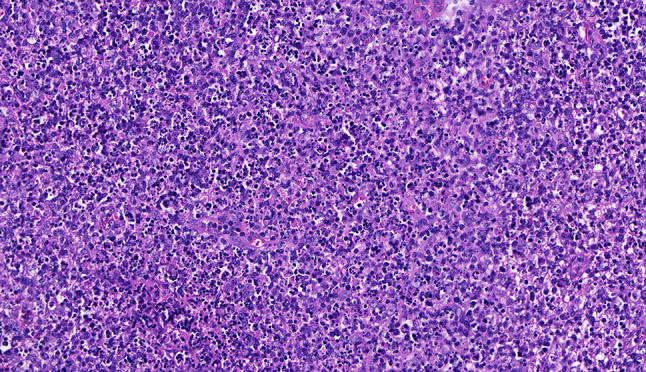
Postoperative paraffin of right cervical lymph node section: Irregular faintly stained lesions in lymph nodes: numerous nuclear fragments, mononuclear-like histiocytes and plasmacytoid dendritic cell proliferation, scattered fibrin deposits, no neutrophils (hematoxylin and eosin, original magnification, x 400)

**Fig. 6:**
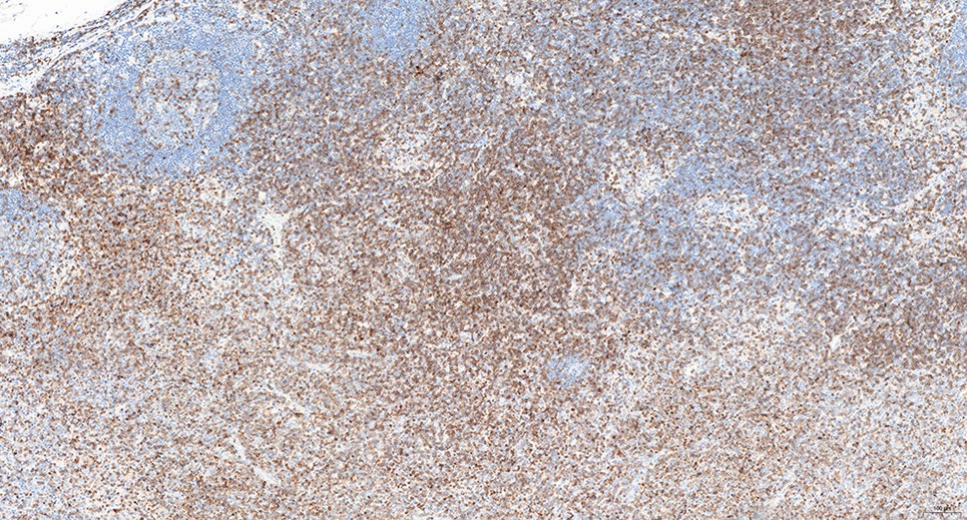
Postoperative immunohistochemical section of the right cervical lymph node in paraffin: the faintly stained lesion positive for CD3. Envision method x 50

**Fig. 7:**
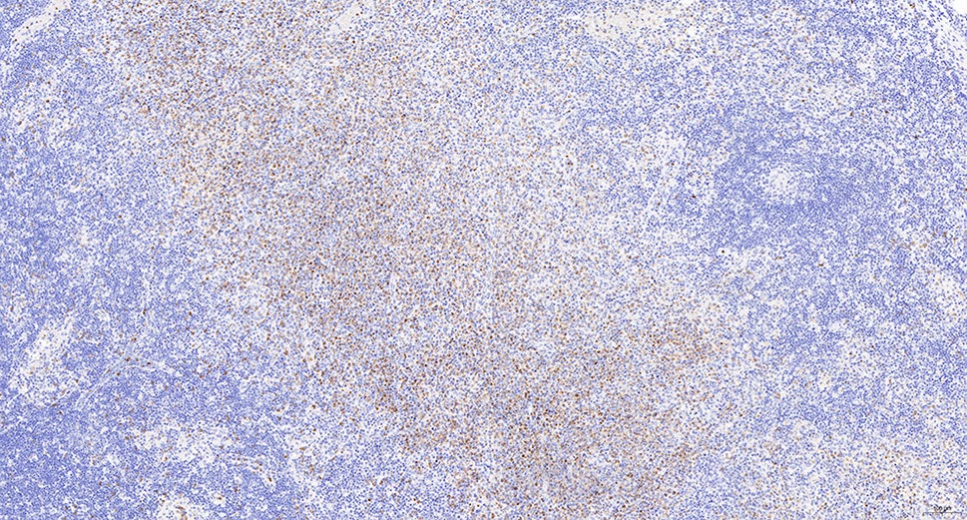
Postoperative immunohistochemical section of the right cervical lymph node in paraffin: the faintly stained lesion positive for MPO. Envision method x 50

**Fig. 8:**
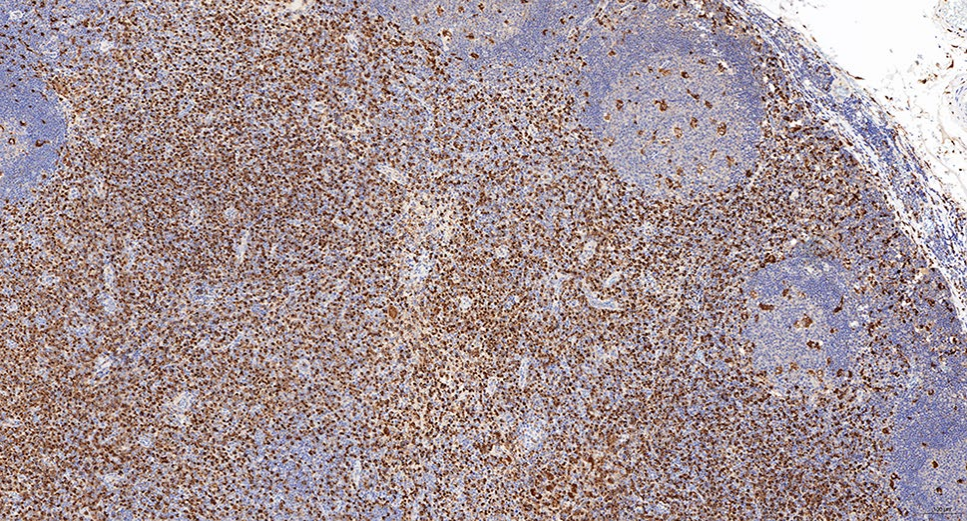
Postoperative immunohistochemical section of the right cervical lymph node in paraffin: the faintly stained lesion positive for CD68. Envision method x 50

**Fig. 9:**
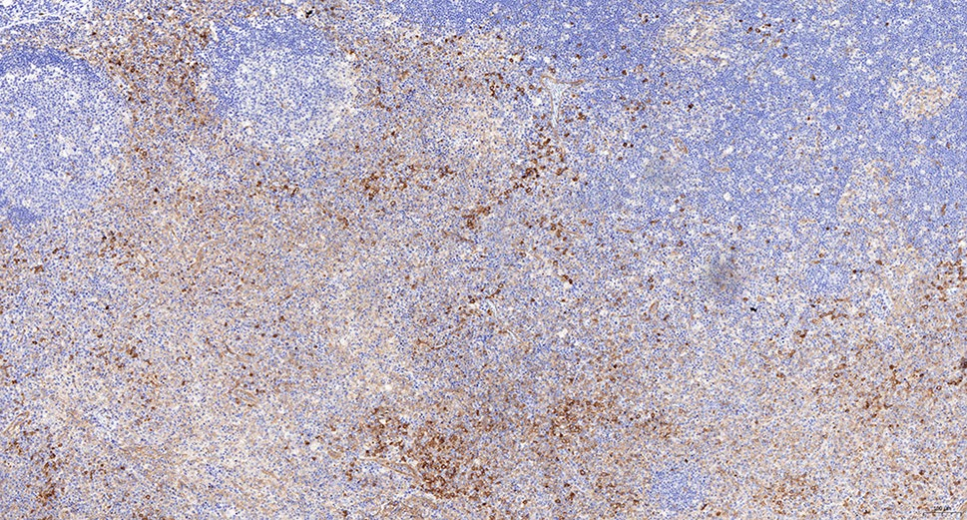
Postoperative immunohistochemical section of the right cervical lymph node in paraffin: the faintly stained lesion positive for CD123. Envision method x 50

**Fig. 10:**
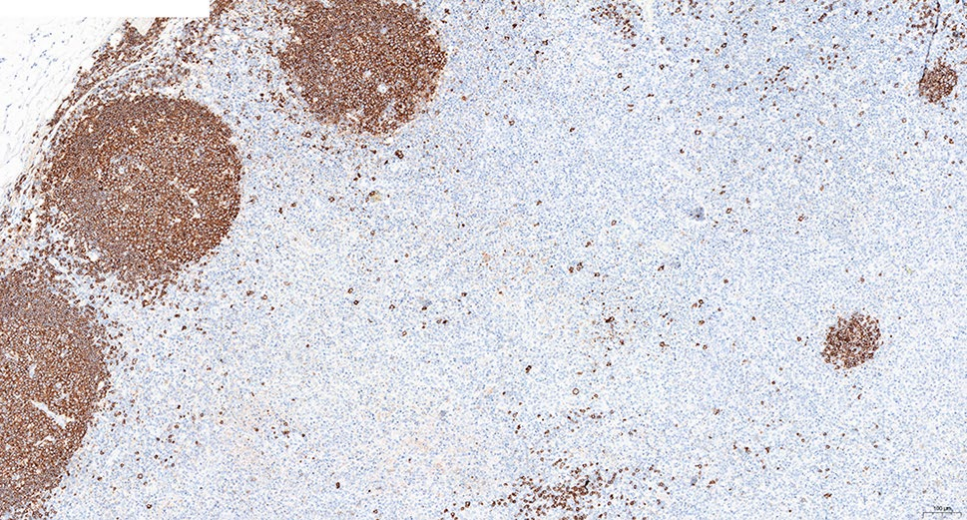
Postoperative immunohistochemical section of the right cervical lymph node in paraffin: scattered positivity for the faintly stained lesion CD20. Envision method x 50

**Fig. 11:**
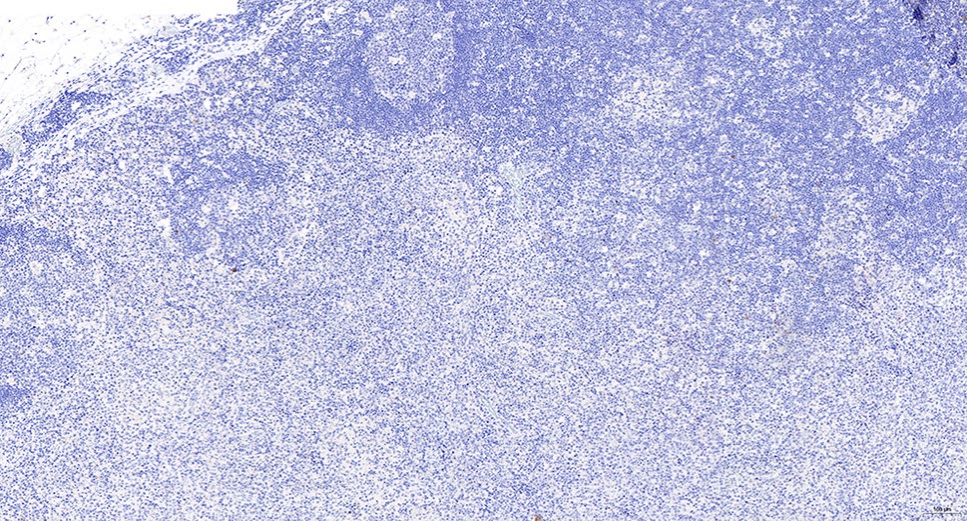
Postoperative immunohistochemical section of the right cervical lymph node in paraffin: the faintly stained lesion negative for CD1a. Envision method x 50

**Fig. 12:**
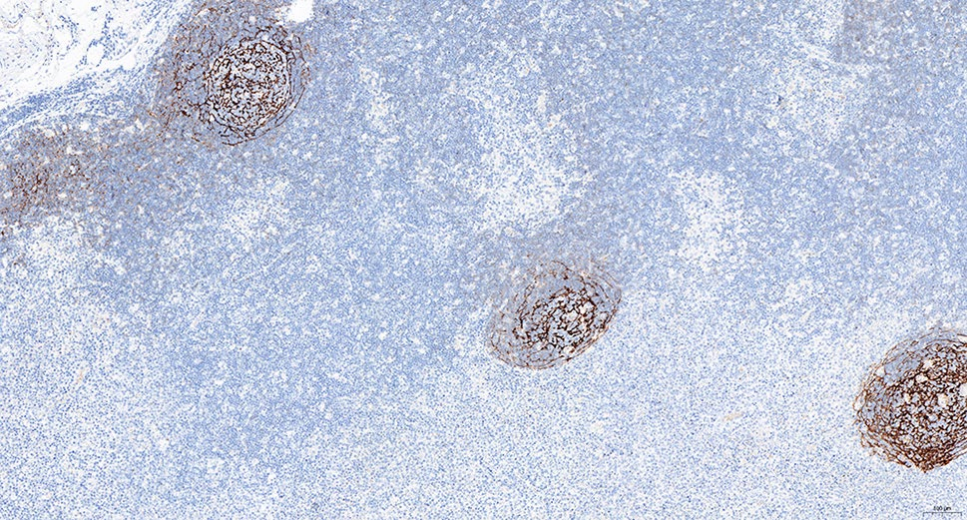
Postoperative immunohistochemical section of the right cervical lymph node in paraffin: the residual FDC network expressed CD21. Envision method x 50

**Fig. 13:**
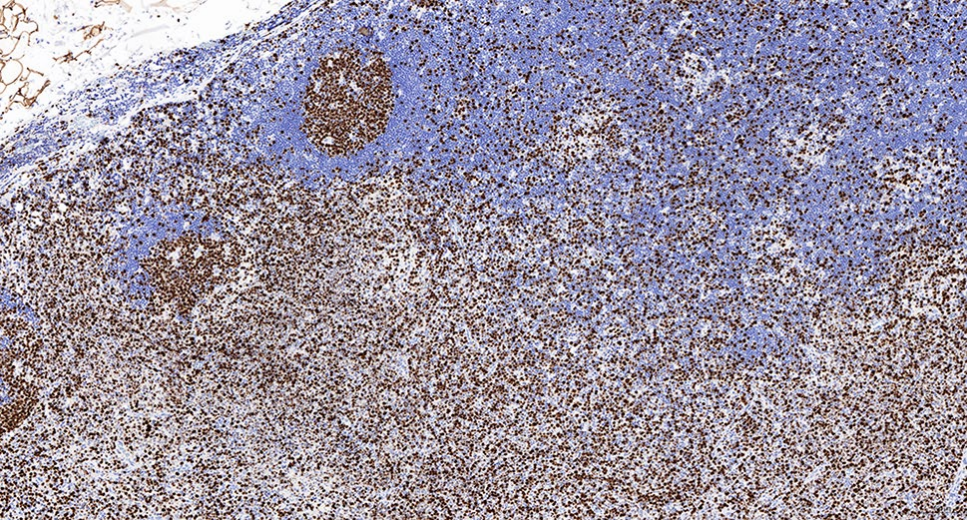
Postoperative immunohistochemical section of the right cervical lymph node in paraffin: Ki67 was highly expressed in faintly stained lesions and germinal centers. Envision method x 50

**Fig. 14:**
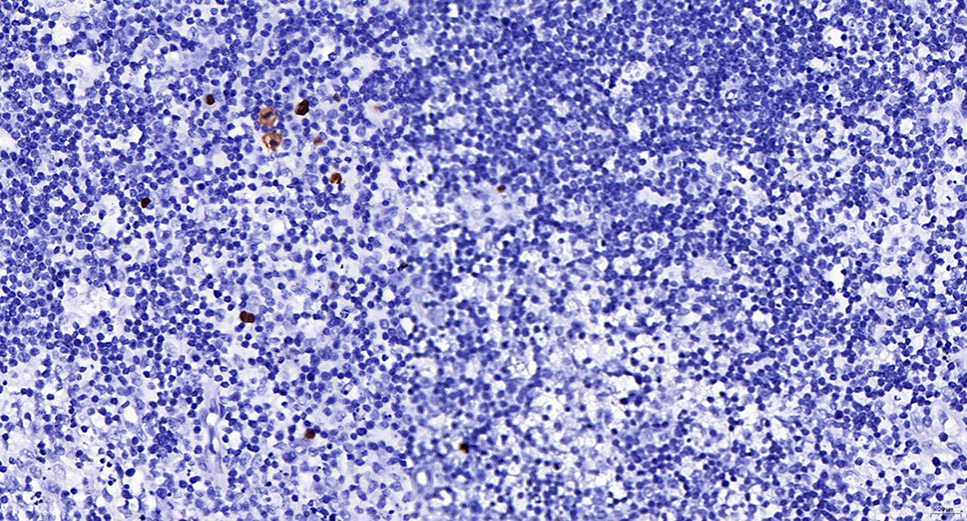
Postoperative in situ hybridization of EBV-EBER in the right cervical lymph nodes in paraffin: scattered positive faintly stained lesions. In situ hybridization method x 400

**Fig. 15:**
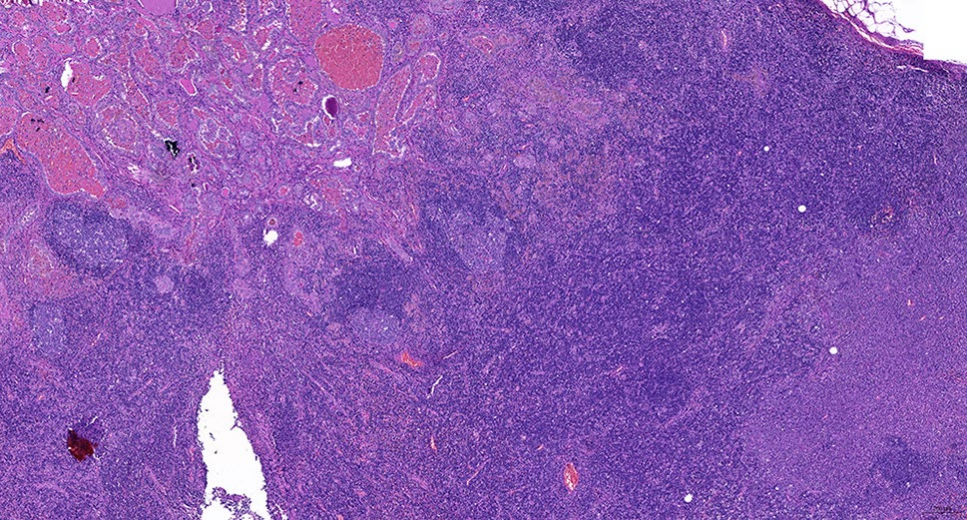
Postoperative paraffin section: The coexistence of histiocytic necrotizing lymphadenitis and metastatic PTC was found in the same lymph node. HE X 50

**Fig. 16:**
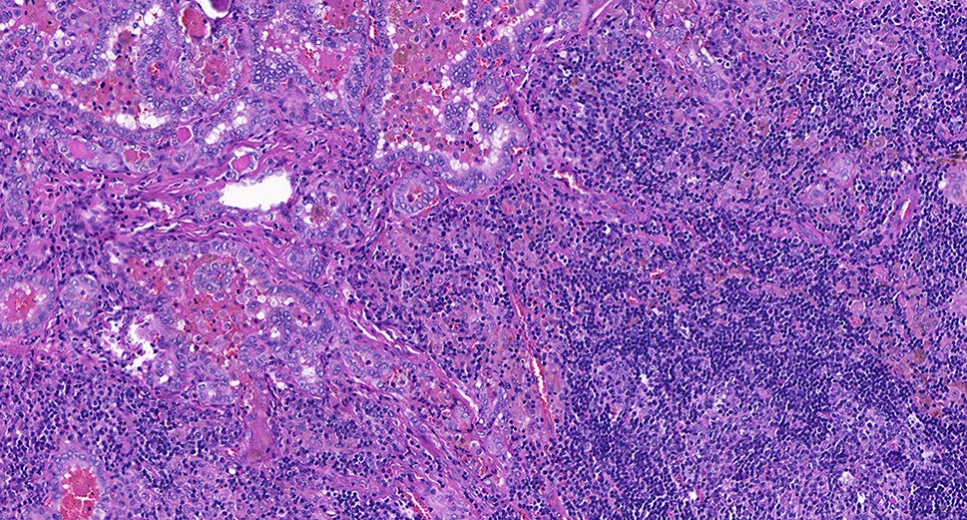
Postoperative paraffin section: Papillary thyroid cancer in coexisting lymph nodes. HE x 400

**Fig. 17:**
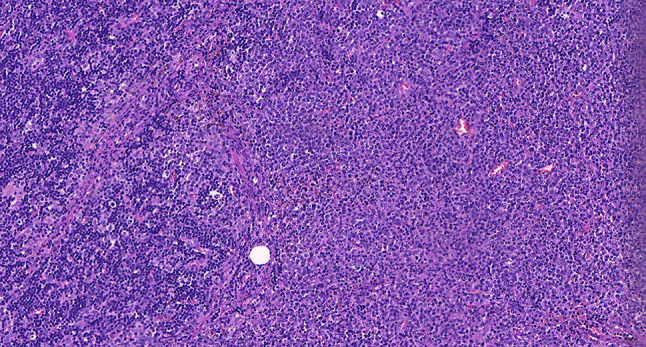
Postoperative paraffin section: Histiocytic necrotizing lymphadenitis in coexisting lymph nodes. HE x 400

Intraoperative freezing for inspection: Left thyroid gland, about 4.5 × 3 × 2 cm in size, two gray‒white areas with diameters of 0.2 and 0.3 cm were seen on the section, respectively, soft. Right thyroid gland, approximately 4.5 × 3 × 2 cm in size, a grayish white nodule, with a size of 1.2 × 0.9 × 0.8 cm, immediately adjacent to the capsule, and another grayish yellow nodule, 0.2 cm from the capsule, with a diameter of 0. 2 cm, both hard. The frozen section report was given: (left thyroid) benign lesion. (Right thyroid) Papillary thyroid carcinoma. Then, right neck dissection was performed. Postoperative paraffin pathology was shown: (right thyroid) Papillary thyroid carcinoma (diffuse sclerosing variant), invaded with the capsule (Fig. 4). Typical metastatic papillary thyroid carcinoma was seen in some lymph nodes, and some lymph nodes showed focal irregular pale pink stained lesion areas in cortical and paracortical areas with numerous nuclear fragments, the proliferation of mononuclear-like histiocytes and plasmacytoid dendritic cells. Coagulative necrosis was seen focally, scattered cellulose deposition, few plasma cells, and no neutrophils were seen (Fig. 5). The results of the immunohistochemical staining showed CD3, MPO and CD68 were expressed in most of the cells in the pale pink stained lesion areas. The expression of CD123 was slightly less than that of the previous antibodies. And CD20 was expressed sporadically (Figs. 6–10). But CD1a was not expressed (Fig. 11). CD21 showed a residual follicular dendritic cell network (Fig. 12), Ki67 was highly expressed in pale pink stained lesion areas and germinal centers (Fig. 13). Epstein‒Barr virus was detected by Epstein‒Barr encoding region (EBER) in situ hybridization. EBER was scattered and positive in the pale pink stained areas (Fig. 14). Interestingly, the coexistence of pale pink stained lesions and metastatic PTC was found in the same lymph node (Figs. 15–17).

Pathological diagnosis was given: (left) nodular goiter, (right) papillary thyroid carcinoma (diffuse sclerosing variant) (two foci, maximal diameter approximately 1.2 cm and 0.2 cm), the capsule was invaded; metastatic PTC was found in 16 of 57 lymph nodes on the right side of the neck (the maximal diameter of metastatic lesions was 1.8 cm), and some lymph node biopsies showed histiocytic necrotizing lymphadenitis. Three lymph nodes were seen with histiocytic necrotizing lymphadenitis coexisting with metastatic PTC. However, there was no metastatic PTC or HNL in the left cervical or prelaryngeal lymph nodes.

Follow-up: The patient recovered well after surgery and survived disease-free for more than 5 months, and the long-term prognosis remains to be observed further.

## Discussion

The clinical manifestations of the HNL lack specificity and may resolve spontaneously within 1 to 6 months after diagnosis. The most common manifestation of patients is localized cervical lymph node enlargement with tenderness, often accompanied by fever. Other rare symptoms include vomiting, diarrhea, night sweats, upper respiratory symptoms, etc. The disease is rare in extranodal lymph nodes, and is most common in the skin, where skin involvement usually presents as rashes, nodules, erythematous papules and erythema multiforme on the face and upper trunk [2]. It also rarely occurs in the bone marrow, liver, submandibular glands [3] and parotid glands [4]. The histological features are patchy and irregular necrotic areas with the expansion of the paracortical area of lymph nodes. Apoptotic bodies, crescent tissue cells, and proliferating plasmacytoid monocytes are seen in the necrotic area, accompanied by abundant nuclear fragments but a lack of neutrophils and eosinophils. According to the different stages of the disease, it is divided into three types: the proliferative type, the necrotizing type and the xanthomatous type. The proliferative type is characterized by the proliferation of histiocytes and plasmacytoid dendritic cells, mixed with small lymphocytes and nuclear fragmentation, while necrosis is rare or absent; the necrotizing type is the most common and is characterized by a significant increase in necrotic components; and the xanthomatous type refers to the predominance of foamy histiocytes in the lesion [5]. This case is the necrotizing type. The disease is self-limiting, and no specific treatment is recommended. The treatment is aimed at relieving symptoms (rest, analgesics and antipyretics) and corticosteroids are available for recurrent disease or for patients with a more severe clinical course [4]. There are no definitive laboratory tests to diagnose HNL, and lymph node biopsy should be performed in persons suspected of this disease to avoid misdiagnosis.

**Table 1:** Summary of cases of HNL combined with other tumors in the literature

Number	Year of publication	Author Nationality	Sex	Age	Concomitant tumor	Fever or not	Lymph node metastasis tumor and number	Merge HNL and number of	Treatment	Recurrence or not	prognosis
1	1997	Canada	Male	37	Signet ring cell carcinoma of esophageal-gastric	-	NA	cervical lymph node	Esophageal-gastrectomy	NA	Death by extensive peritoneal and mediastinal metastases
2	2000	Britain	Fe	66	carcinoma of breast	-	0/13	Axillary lymph nodes 12 / 13	Breast cancer radical surgery	-	Symptoms improved
3	2015	Korea	Ma	38	Right PTC	+	Several on the right	One right cervical lymph node	Total thyroidectomy and central neck dissection, postoperative I131, methylprednisolone and NSAIDs	-	Symptoms improved
4	2015	America	Fe	30	Right PTC	+	One on the right	Other lymph nodes in the right neck	Total thyroidectomy and bilateral central and right lymph node dissection	-	Symptoms improved
5	2015	Canada	Fe	37	Melanoma of the thigh	-	1/10	Five left inguinal lymph nodes	Surgical excision	-	NA
6	2017	Japan	Ma	48	Squamous cell carcinoma of the tongue	NA	2	Right posterior neck lymph node	Surgical excision	+	Symptoms improved
7	2023	China	Ma	48	Right PTC	-	16/57	Three right cervical lymph nodes	Radical thyroidectomy, right functional neck lymph node dissection and right upper mediastinal lymph node biopsy were performed	-	Symptoms improved
8	1987	Hong Kong	Fe	57	Recurrent malignant fibrous histiocytoma of thigh	NA	There were no tumor metastases	Left inguinal lymph node	Extensive tumor resection	-	Symptoms improved
9	2000	Japan	Fe	27	Diffuse Large B-cell lymphoma in remission	-	/	Left side of the cervical lymph node	No treatments	-	Symptoms improved
10	2000	Japan	Fe	30	Diffuse Large B-cell lymphoma in remission	-	/	Left side of the axillary lymph node	/	-	Symptoms improved
11	2021	Britain	Fe	47	Multiple myeloma	+	NA	Left side of the axillary lymph node	Steroid treatment	-	Symptoms improved

*Abbreviations: Fe: Female. Ma: male. NA: not available

The pathogenesis of HNL is still unclear. It is assumed that HNL represents the T-cell mediated immune response of genetically susceptible populations to various antigens, and patients with HNL more often have specific human leukocyte antigen (HLA) Class II alleles, specifically HLA-DPA1 and HLA-DPB1, compared with the general population. These alleles are more prevalent in Asians and extremely rare or absent in whites, which may account for the disease being more common in Asians. Pathogens associated with triggering this response include Epstein‒Barr virus, human herpes virus, microvirus B19, cytomegalovirus, and human herpesvirus. HNL can be associated with autoimmune diseases such as systemic lupus erythematosus, mixed connective tissue disease, psoriasis and other autoimmune diseases, suggesting that it may be a potential manifestation of autoimmune disease [6].

HNL needs to be differentiated from lymphoma, infectious lymphadenitis, systemic lupus erythematosus, infectious mononucleosis, and other diseases. (1) The proliferation of immunoblasts and plasmacytoid dendritic cells at the margins of HNL necrotic foci can mimic the invasion of T cells or B cells of non-Hodgkin’s lymphoma and be easily confused. However, the tumor cells of lymphoma have obvious cell atypia, increased volume, thickened nuclear membrane, increased and enlarged nucleoli, and pathological mitosis, but generally no necrotic lesions. Immunohistochemical staining shows that T cells or B cells are cloning-positive. Focal necrosis, nuclear fragmentation and the histiocytic cells that have engulfed nuclear debris may be present in a small number of lymphomas, especially in T-cell lymphoma. However, positive TCR gene rearrangement, few histiocytic cells, and a long course of disease all support the diagnosis of lymphoma [7]. (2) Necrotizing lymphadenitis can be caused by a variety of infectious factors and is easily confused with HNL. Epithelioid histiocytosis with granulomatous formation and scattered giant cells are seen in necrotizing lymphadenitis of tuberculosis, histoplasmosis, leprosy, and cat-scratch disease. In cases of syphilitic necrotizing lymphadenitis, there is usually a prominent perivascular infiltration of plasma cells, while a large number of neutrophils are often present in bacterial infections [5]. Special stains and immunohistochemical stains are helpful in identifying the infectious agents. In our case, the blood culture for acid-fast bacilli of the patient was negative on admission. And the multinucleated giant cells, caseous necrosis and well-formed granulomas were absent, although abundant histiocytes were present. Moreover, Ziehl–Neelsen stain has been done and negative to rule out tuberculosis. (3) The lymph nodes of systemic lupus erythematosus show varying degrees of cortical necrosis, accompanied by nuclear debris and inflammatory cell reactive proliferation. Hematoxylin bodies assist in identification. They are usually located in or near the necrotic foci, but may also be located in the lymphatic sinuses, paracortex or vascular wall.

Clinically abnormal serum immunology, especially positive antinuclear antibodies, is helpful for the diagnosis of systemic lupus erythematosus. (4) Infectious mononucleosis is characterized by interfollicular enlargement, immunoblast proliferation, single-cell apoptosis and necrotic foci are common, and histiocytic and plasmoid dendritic cells are rare [8]. (5) The identification of histiocytic proliferative lesions is also essential. we focus on the most common histiocytosis among adults: Langerhans cell histiocytosis (LCH), Erdheim-Chester disease (ECD) and Rosai-Dorfman disease (RDD). The primary differential diagnosis is Langerhans cell histiocytosis (LCH), LCH lesions often show histiocytes mixed with a significant infiltration of inflammatory cells. And neoplastic LCH cells are mononucleated, typically with a coffee bean-shaped nucleus. Binucleated or multinucleated cells with the typical Langerhans cell cleft can be identified [9]. Moreover, abundant eosinophils are often observed. Characteristic immunohistochemistry such as S-100 and CD1a are helpful for identification. In our case, S-100 and CD1a were negative. And ECD mostly occurs in long tubular bones and is distributed symmetrically. Additionally, the histology of ECD shows infiltration of tissue by small CD1a– mononucleated histiocytes, sometimes associated with Touton cells. Furthermore, the histology of RDD is a massive expansion of histiocytes in the lymph node sinuses with lymphocytes and plasma cells [10]. Abundant plasma cells in the medullary cords and around the venules are typical. In combination with clinic information, histological patterns and immunohistochemistry, the above lesions were excluded.

HNL and papillary thyroid carcinoma coexisting in the same lymph node is uncommon and seldom ever documented, according to a review of the current literature; so far, only two cases have been retrieved [11]. At present, 10 cases of HNL combined with other tumors have been reported (Table 1), most of which occurred in women (7/11), predominantly in Asia (6/11), aged 27–66 years. The tumors combined with HNL were PTC [6, 11] (2 cases), gastric carcinoma [12] (1 case), breast carcinoma [13] (1 case), squamous cell carcinoma of the tongue [14] (1 case), malignant melanoma [15] (1 case), malignant fibrous histiocytoma [16] (1 case), multiple myeloma [17] (1 case), and diffuse large B lymphoma in remission [18] (2 cases).

It can be accompanied by fever or no fever, generally without special treatment, and steroids and other hormones can be used for symptomatic treatment. Recurrence is rare (1/11); treatment of the tumor is the main focus when there is tumor coexistence. HNL coexisted with tumors: Cases 1 to 7, similar to our case, were evaluated preoperatively as metastatic tumors of the lymph node, and the HNL occurred on the same side of the tumor. In cases 8–11, lymph nodes were enlarged months or years after tumor treatment, and the biopsy was KFD.

Review the literature on the coexistence of HNL with other tumors: PTC [6, 11], gastric carcinoma [12], breast carcinoma [13], squamous cell carcinoma of the tongue [14], and malignant melanoma [15], all of which occurred on the same side of the tumor as the present case, may indicate that HNL can be induced by tumor-associated local antigens and raise the possibility of specific immune responses to antigenic stimulation in HNL. Dequante et al. reported a possible correlation between increased cytotoxic activity of T cells stimulated by the tumor and disease transformation [17]. Apoptosis of target cells is induced by two molecular mechanisms of T-cell-mediated cytotoxicity, one perforin-based and the other Fas-based [19]. The reason for the simultaneous occurrence of this case may be closely related to the patient’s infection with Epstein‒Barr virus. We speculate that EBV infection of lymph nodes activates a variety of cells, including T cells and histiocytes, and promotes massive T cell proliferation. Activated histiocytes produce various cytokines that, through Fas and FasL interaction, induce apoptosis of T cells.

We speculate that EBV infection of lymph nodes activates a variety of cells, including T cells and histiocytes, and promotes massive proliferation of T cells. Activated histiocytes produce various cytokines that, through Fas and Fasl interaction, induce apoptosis of T cells.

Moreover, FASL was highly expressed in papillary thyroid carcinoma [20], suggesting that papillary thyroid carcinoma may increase the cytotoxic activity of T cells and the specific immune response of its own HNL, but there is not enough evidence to show whether this is a causal relationship, and more experiments and data are needed to prove it.

## Conclusion

The coexistence of HNL and papillary thyroid carcinoma is unique, and the coexistence of the two diseases is rare, but the reason why the associated diseases can coexist has not been proven. When patients present with enlarged lymph nodes in the neck, they should be considered as a differential diagnosis. The pathological diagnostician should not only focus on the tumor metastasis in the lymph nodes but also pay attention to the inflammatory lesions in the lymph nodes, such as HNL and Castleman, because these lesions may mislead the clinical staging of the tumor by the clinical doctor and lead to unnecessary treatment.

## Data Availability

All data generated or analyzed in this study are included in this article.

## Abbreviations

HNL: Histiocytic necrotizing lymphadenitis
KFD: Kikuchi-Fujimoto disease
CDFI: Color Doppler flow imaging
TI-RADS: Thyroid Imaging Reporting and Data System
